# Thermal Conductivity and Electrical Resistivity of Melt-Mixed Polypropylene Composites Containing Mixtures of Carbon-Based Fillers

**DOI:** 10.3390/polym11061073

**Published:** 2019-06-21

**Authors:** Beate Krause, Piotr Rzeczkowski, Petra Pötschke

**Affiliations:** Leibniz-Institut für Polymerforschung Dresden e.V., Hohe Str. 6, 01069 Dresden, Germany; krause-beate@ipfdd.de (B.K.); rzeczkowski@ipfdd.de (P.R.)

**Keywords:** thermal properties, electrical properties, polymer–matrix composites (PMCs), carbon nanotubes

## Abstract

Melt-mixed composites based on polypropylene (PP) with various carbon-based fillers were investigated with regard to their thermal conductivity and electrical resistivity. The composites were filled with up to three fillers by selecting combinations of graphite nanoplatelets (GNP), carbon fibers (CF), carbon nanotubes (CNT), carbon black (CB), and graphite (G) at a constant filler content of 7.5 vol%. The thermal conductivity of PP (0.26 W/(m·K)) improved most using graphite nanoplatelets, whereas electrical resistivity was the lowest when using multiwalled CNT. Synergistic effects could be observed for different filler combinations. The PP composite, which contains a mixture of GNP, CNT, and highly structured CB, simultaneously had high thermal conductivity (0.5 W/(m·K)) and the lowest electrical volume resistivity (4 Ohm·cm).

## 1. Introduction

In order to achieve materials with high thermal conductivity, composites based on thermoplastics and thermally conductive fillers are an interesting alternative to metals. Thereby, the low price, low density, and good processability by melt extrusion and injection molding are advantages of thermoplastic polymers. Such composite materials can be used e.g., in heat exchangers or geothermal systems [1,2]. These fillers have to form a thermally conductive network in the matrix in order to transfer their high conductivity into the composite. Next to high thermal conductivity, some applications require at the same time also high electrical conductivity, such as bipolar plates in fuel cells [3,4,5]. The electrical conductivity requires networks with neighboring conductive particles, which can be separated by thin polymer films with distances below the electron hopping and or tunneling distance (assumed to be around 2–8 nm) [6]. Only the formation of an electrical network at the percolation threshold concentration changes the electrical properties from insulating to electrically conductive [7,8]. In contrast, the thermal conductivity requires phonon transport between neighboring thermal conductive fillers. Thus, the trends in the development of electrical properties and thermal conductivity with filler content are very different for polymer composites [9,10,11,12,13,14,15,16,17,18,19,20,21].

Carbon-based materials such as highly thermal conductive carbon nanotubes (CNT) [12,22], graphite (G) [17], carbon fibers (CF), carbon black (CB), or graphite nanoplatelets (GNP) [23] appear to be the best fillers to couple high thermal as well as electrical conductivity with light weight. In current research, the trend of improving the thermal conductivity of polymers is focused on the use of nanofillers with high thermal conductivity [22].

However, the huge interface in nanocomposites together with the large thermal resistance between filler surfaces and the surrounding polymer matrix hinders the transfer of phonons over these interfaces. Thus, despite the exceptionally high intrinsic thermal conductivity of CNT [1,10,24,25,26,27,28,29,30,31], relatively low thermal conductivities of polymer/CNT nanocomposites were observed experimentally. One possible way to promote the formation of thermally conductive pathways is the combination of different types of fillers with different dimensions and/or shapes [1,27,32,33,34,35]. Synergistic effects are expected in such composites, which means that the effect caused by the use of the hybrid filler system is greater than the overall effect of the individual fillers. When using anisotropic fillers, a thermal conductivity dependent on the measuring direction is to be expected due to the orientation and alignment processes of the fillers when shaping test specimens. In most cases, the thermal conductivity along fibers or platelet-like fillers is greater than perpendicular to this direction. Since processing usually produces structures with an orientation in the processing direction (parallel to the surface), in plate-shaped samples, the conductivity is typically higher in plane (along) than through the sample. When comparing values for thermal conductivity, it also has to be considered that different measuring principles may result in different absolute values. Therefore, only a comparison with the value of the base material is meaningful. 

When using 7.5 vol% multiwalled CNT (MWCNT) in polybutylene terephthalate (PBT), Pflug et al. [27] measured on injection molded specimens a thermal conductivity of 0.43 W/(m·K) perpendicular ⊥ and 0.59 W/(m·K) parallel ║ to the injection direction. Compared to the unfilled PBT, this represents increases up to 170% and 236%, respectively. The authors found synergistic effects for the combination of MWCNT with iron powder in such PBT composites. While the composites with only one filler achieved thermal conductivities of 0.3 W/(m·K) ⊥ or 0.5 W/(m·K) ║ (PBT/4 vol% MWCNT) and 0.38 W/(m·K) ⊥ or 0.43 W/(m·K) ║ (PBT/10 vol% iron), for the three-component system (PBT/5 vol% MWCNT + 10 vol% iron), thermal conductivities of 0.55 W/(m·K) ⊥ and 0.94 W/(m·K) ║were determined. In addition, they reported also synergism for high-density polyethylene (PE-HD) composites with 5 vol% MWCNT and 60 vol% aluminum oxide. Mazov et al. [33] reported synergistic effects for the thermal conductivity of melt-mixed polypropylene (PP) composites filled with CNT and CF, which were shaped by injection molding. The addition of 4 wt% CNT to PP (0.23 W/(m·K) ║, ⊥) led to a value of 0.34 W/(m·K) ║, ⊥ and the incorporation of 40 wt% CF to 1.23 W/(m·K) ║ and 0.55 W/(m·K) ⊥. The PP composite containing the mixture of 4 wt% CNT and 36 wt% CF resulted in values of 1.9 W/(m·K) ║ and 0.9 W/(m·K) ⊥. This effect was caused by the longitudinal CF alignment and their crosslinking by CNT. In PE-based melt-mixed composites, Müller et al. [1] found no enhancement of the thermal conductivity (⊥) for mixtures of MWCNT (Nanocyl^TM^ NC7000) and expanded graphite (EG) (0.63 W/(m·K) @ PE/5 wt% MWCNT + 5 wt% EG) as compared to the use of the EG as a single filler (0.74 W/(m·K) @ 10 wt% EG). Interestingly, the addition of 10 wt% of microsilica with low thermal conductivity to a composite with 5 wt% CF resulted in an enhancement in thermal conductivity from 0.61 W/(m·K) toward 0.71 W/(m·K) [1]. This effect was explained by the low-conductivity microsilica supporting the formation of the conductive CF network. Mixed fillers of MWCNT and boron nitride (BN) or MWCNT and synthetic diamond (SD) at a constant total filler content of 4 vol% in PP were studied by Nurul et al. [36]. It was found that the thermal conductivity (⊥) increases from 0.22 W/(m·K) (pure PP) with increasing MWCNT content and simultaneously decreasing BN or SD content up to 0.35 W/(m·K) at PP/4 vol% MWCNT, whereas the thermal conductivity of PP/4 vol% SD and PP/4 vol% BN were measured to be 0.33 W/(m·K) and 0.27 W/(m·K), respectively. The values of the PP composites with mixed filler systems lie between these two values of the composites with single fillers. Thus, no synergy is discernible.

Concerning the electrical percolation threshold of mixed carbon filler systems, Sun et al. [37] developed an equation for the theoretical percolation threshold of mixed fillers based on the percolation of the single fillers by adapting an excluded volume approach. If the experimental threshold is found to be lower than the calculated one, it can be assumed that the system shows a synergistic effect. Such synergistic effects have been successfully demonstrated for the electrical conductivity of epoxy [38,39] and thermoplastic polymers [40,41,42,43,44] filled with carbon nanotubes and carbon black. Naji et al. [19] described for polycarbonate (PC) composites that CNT can act as a bridge between larger fillers, such as carbon fibers and graphite, and fill the gaps to form a more effective conductive network. There are only a few papers that consider the electrical and thermal conductivity of polymer nanocomposites together [11,13,17,19,21].

For the formation of the percolated network, the aspect ratio—i.e., the length to diameter ratio of filler—is of particular importance. If the aspect ratio is high, e.g. for carbon nanotubes, a percolated network can be formed at relatively low filler content. With decreasing aspect ratios, e.g. for carbon black, graphite, or graphite nanoplatelets, a higher amount of filler is necessary to achieve electrical percolation [7,8]. 

In order to compare different kinds of carbon black, the structure of its aggregates and particle size have to be taken into account. Highly structured carbon black contains a high number of particles per aggregate. At a given loading, a higher structured CB is expected to result in a higher electrical conductivity of the composite than a low structured CB [45]. However, the formation of a conductive network is also influenced by the dispersibility of CB, which is better for highly structured carbon black. To characterize the CB structure, the oil adsorption number (OAN) is a typical measure, and higher values are characteristic for highly structured carbon black.

In this study, composite materials based on polypropylene (PP) containing different kinds of carbon-based fillers were prepared using small-scale melt mixing compounding. This investigation focused on PP, as it is a widely used material for bipolar plate applications [2,5,17,21] where both electrical and thermal conductivity play a role. In order to see general effects and enable the desired good melt processability, the filler content was limited to 7.5 vol%. Graphite nanoplatelets (GNP), carbon fibers (CF), multiwalled carbon nanotubes (MWCNT), carbon black (CB), and graphite (G) were used, as well as mixtures of these fillers. The amounts and mixing ratios of the hybrid filler systems were varied in order to determine the influence on the achievable level of thermal and electrical conductivity. Especially, mixtures of fillers with different shapes, particle sizes, and aspect ratios were investigated to gain knowledge about the effects of mixed filler networks. The goal is to achieve a composite having simultaneously high thermal and high electrical conductivity at the selected loading.

## 2. Materials and Methods

Polypropylene (PP) Moplen HP400R (LyondellBasell Industries, Rotterdam, The Netherland) was applied as a matrix having a melt flow rate of 25 g/10 min @ 230 °C and 2.16 kg and a granular shape. 

For the filler, the following were used:

As carbon fibers (CF): SIGRAFIL^®^ C25 M250 UNS Milled Carbon Fiber (SGL Carbon Group, Wiesbaden, Germany) was used with a carbon content of >95 wt %, mean fiber length of 0.135 mm, fiber diameter of 7.5 µm, density of 1.8 g/cm^3^, and a bulk density of 0.23 g/cm^3^.

As graphite nanoplatelets (GNP): xGNP-M-5 (Graphene Nanoplates from XG Sciences, Inc., Lansing, MI, USA) were used with a layer thickness of 6–8 nm, particle size of 5 µm, surface area of 120–150 m^2^/g, carbon content of >99.5%, density of 2.2 g/cm^3^, bulk density of 0.03–0.1 g/cm^3^, and thermal conductivity of 3000 W/(m·K) parallel to the surface.

As multiwalled carbon nanotubes (MWCNT): NC7000 (Nanocyl^TM^, Sambreville, Belgium), produced in an industrial large-scale catalytic vapor deposition process, with an average diameter of 10 nm, average length of 1.3 µm [46], carbon purity of 90%, surface area of 250–300 m²/g [47], and density of 1.75 g/cm^3^ were applied.

Three different kinds of carbon black (CB, Orion Engineered Carbons GmbH, Frankfurt am Main, Germany) with a density of 1.7–1.9 g/cm^3^ were used, namely Printex^®^ XE2B pearled, Printex^®^ L6 pearled, and Printex^®^ 300 pearled. The properties are summarized in Table 1. Additionally, the particle size of the materials was measured (Table 2). The thermal conductivity of CB is around 90 W/(m·K) [22].

Three different kinds of graphite (G) having a density of 2.2 g/cm^3^ were used, which are TIMCAL TIMREX^®^ KS150 (Imerys Graphite & Carbon, Bodio, Switzerland) with a bulk density of 0.42 g/cm^3^, TIMCAL TIMREX® KS500 (Imerys Graphite & Carbon, Bodio, Switzerland) with a bulk density of 0.8 g/cm^3^, and EP1200 (Richard Anton KG, München, Germany). The typical particle sizes, as measured in this study, are summarized in Table 2. The thermal conductivity of graphite is around 250 W/(m·K) [22]. 

For the measurement of the particle size distributions of the dry graphite and carbon black powders, a laser diffraction sensor HELOS/BR combined with the dispersion units RODOS and ASPIROS (Sympatec GmbH, Clausthal-Zellerfeld, Germany) according to ISO 13320 were used. The measurement range was 0.5 to 875 µm.

Melt compounding was performed using a conical twin screw microcompounder DSM15 (Xplore, Sittard, The Netherlands) at 210 °C and a rotation speed of 150 rpm for 5 min of mixing time. The carbon fillers were dried at 80 °C in a vacuum oven overnight. The fillers and the PP granules were pre-mixed. For the measurements of electrical resistivity and thermal conductivity, the extruded composites were compression molded at 210 °C for 2 min using the hot press PW40EH (Otto-Paul-Weber GmbH, Remshalden, Germany). 

To perform electrical resistivity measurements on compression molded strips (dimension 30 mm x 5 mm x 0.5 mm, cut from pressed plates), a four-point test fixture (gold contact wires with a distance of 16 mm between the source electrodes and 10 mm between the measuring electrodes) combined with a electrometer 6517A (Keithley, Cleveland, OH, USA) or a multimeter DMM 2000 (Keithley, Cleveland, OH, USA) was used. For electrical resistivity values higher than 10^7^ Ohm cm, a Keithley 8009 Resistivity Test Fixture (Keithley, Cleveland, OH, USA) based on ring electrodes was used, and the measurement was done on the compression-molded circular plates (60-mm diameter, 0.5-mm thickness).

The measurement of the thermal conductivity was performed on compression-molded plates (diameter 12.5 mm, thickness 2 mm) through the plates (⊥) using the light flash apparatus LFA 447 NanoFlash (Netzsch-Gerätebau GmbH, Selb, Germany) at 25 °C. A measurement parallel to the pressing direction (║) is not possible.

Morphological characterization of the composites was performed using scanning electron microscopy (SEM) by means of an Ultra plus microscope (Carl Zeiss GmbH, Jena, Germany, field emission cathode) using 3 keV acceleration voltage and an SE2 detector. The composite strands were cryofractured in liquid nitrogen, sputtered with 3-nm platinum, and the surfaces were studied.

## 3. Results

### 3.1. Single Filler Systems in PP

**Figure 1:**
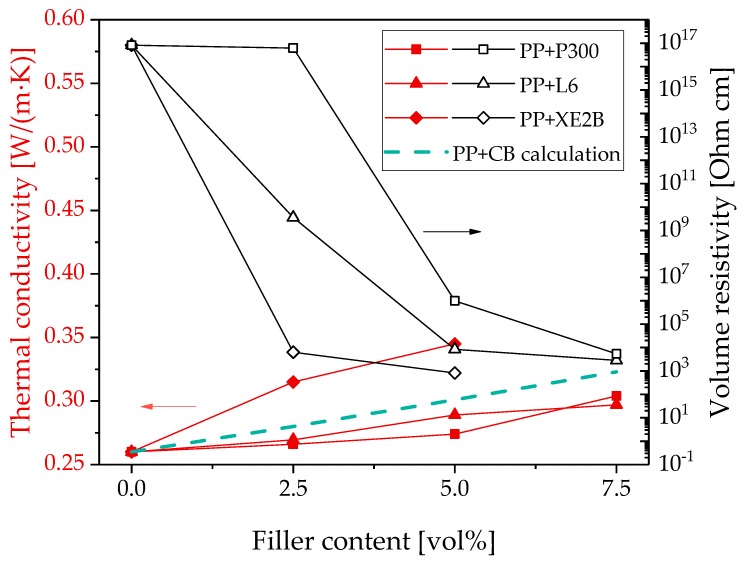
Thermal conductivity and electrical volume resistivity of polypropylene (PP) filled with different kinds of carbon black; values calculated using the Hatta model [48].

**Figure 2:**
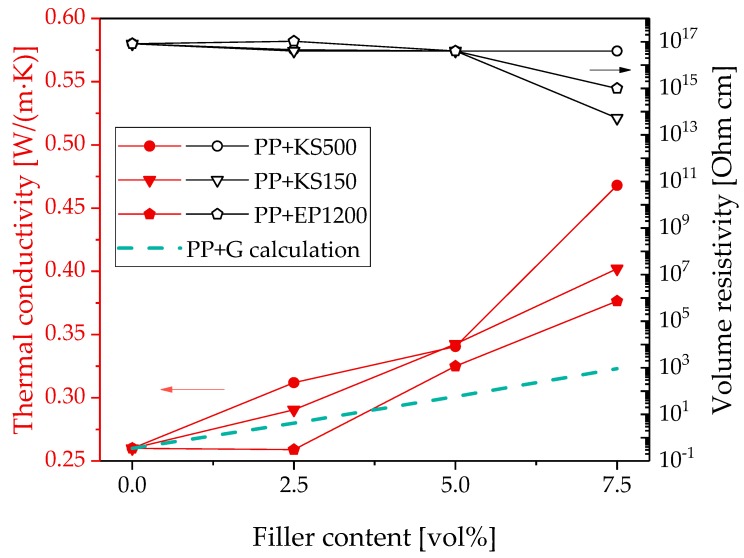
Thermal conductivity and electrical volume resistivity of PP filled with different kinds of graphite; values calculated using the Hatta model [48].

**Figure 3:**
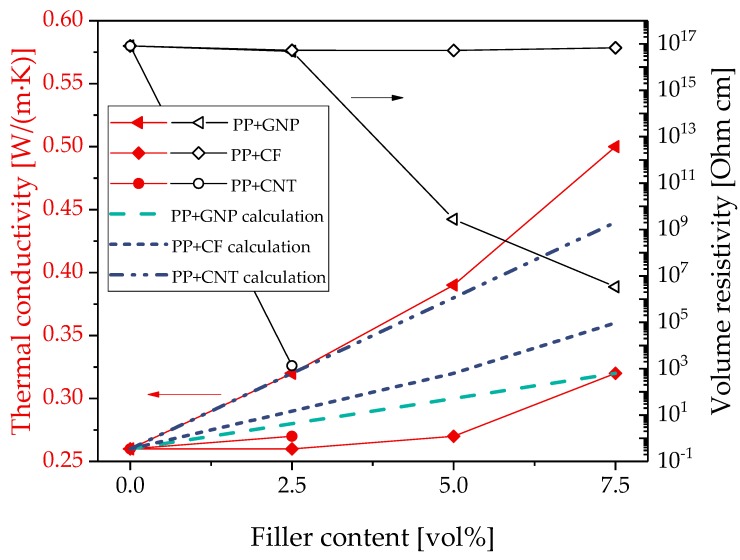
Thermal conductivity and electrical volume resistivity of PP filled with graphite nanoplates, carbon nanotubes, and carbon fibers, calculated values of PP/GNP (graphite nanoplatelets) using the Hatta model (green line) [48] and of PP/CF (carbon fibers) and PP/CNT (carbon nanotubes) using the Xue model (blue lines) [50].

The PP composites containing only one filler type were studied up to filler contents of 7.5 vol%. In Figure 1, Figure 2 and Figure 3, the results of thermal conductivity and volume resistivity are summarized. In connection with the results shown here, an extensive screening for suitable fillers was carried out. For this purpose, PP composites with 5 vol% filler were produced using different filler types, and both the electrical volume resistivity and the thermal conductivity were measured. These results are summarized in Table S1 (PP/carbon black), Table S2 (PP/expanded graphite), and Table S3 part 1–3 (PP/graphite).

The comparison of the different kinds of carbon black shows that the use of the highly structured carbon black (Printex^®^ XE2B) leads to the highest increase of thermal conductivity and the lowest electrical percolation threshold (Figure 1). 

For PP/graphite composites, the highest thermal conductivity value was achieved using graphite KS500 (Figure 2). Compared to the other kinds of graphite, KS500 has the largest particle size. For the electrical properties, the selection of graphite with a lower smaller particle size (KS150) seems to be more effective to achieve a lower volume of resistivity of the PP composites; however, a significant decrease was only achieved at 7.5 vol%. The composites with graphite of the largest particle size (KS500) remained at the value for pure PP even at 7.5 vol% loading.

The comparison of the PP/CB composites with the PP/graphite composites shows that the electrical percolation threshold with the filler CB is significantly lower than that with graphite. However, higher thermal conductivities can be achieved with graphite as the filler.

The results of thermal conductivity and electrical volume resistivity of PP filled with GNP, CF, or CNT are summarized in Figure 3. When comparing graphite and CB-filled composites with those using GNP, CNT, and CF, the highest value of the thermal conductivity was determined for PP/GNP composites. With 0.5 W/(m·K) at 7.5 vol% loading, almost twice the original PP value was achieved. PP/CF composites showed an increase slightly lower than the PP/CB composites with XE2B, but in the range of the other two composite series with CBs. Composites with CNT were only investigated at 2.5 vol%, and the thermal conductivity values is slightly higher than for the CF-containing composite, but lower than that containing GNP. In this comparison, the lowest value of electrical volume resistivity was measured for the PP/2.5 vol% CNT composite (Figure 3). For PP/GNP composites starting at 5.0 vol% loading, a drop in the electrical volume resistivity was found with the electrical percolation threshold being higher than for PP/CB composites (Figure 3). PP/CF composites remain electrically insulating up to 7.5 vol% loading. This may be due to the relatively low aspect ratio of 18 of this kind of CF and corresponds to the findings in ref. [49], in which no significant electrical conductivity up to 20 vol% could be observed when this CF type was added in liquid crystalline polymer.

For the prediction of the thermal conductivity of polymer composites with certain amounts of fillers, calculations based on Hatta model [48] were carried out. The Hatta model is based on Eshelby’s equivalent inclusion model [51], where fillers with the thermal conductivity λ_f_ are substituted by equivalent inclusions that have the same thermal conductivity as the surrounding polymer matrix (λ_m_) and the same eigen-temperature gradient [48]. It can be applied for the prediction of the thermal conductivity λ_c_ of two-component composite systems. Assuming filler particles with a spherical shape (such as carbon black), Equation (1) can be used:

(1) $$\frac{\lambda_{c}}{\lambda_{m}}=1+\frac{V_{f}}{\frac{1}{3}\left(1-V_{f}\right)+\frac{\lambda_{m}}{\lambda_{f}-\lambda_{m}}}$$

where λ_c_, λ_m_, and λ_f_ are the thermal conductivities of the composite, matrix, and filler, respectively, and V_f_ is volume fraction of the filler. The values of the thermal conductivity of each material are given in the experimental section. In a study by Standau et al. [52] on PP-based composites filled with boron nitride particles, the calculated thermal conductivity values were close to the experimental results, suggesting that the Hatta model can provide reliable values. 

For PP/CB composites, the calculated values are in the range of the values measured experimentally, as shown in Figure 1. This illustrates that the model is suitable for CB fillers, even if it is not able to reflect the differences between the differently structured carbon blacks.

For the composites based on the different graphite types, the comparison between the experimental and calculated values of the thermal conductivity shows lower predicted values than measured values (Figure 2). This discrepancy may result from the graphite agglomerate particles not having the assumed spherical shape but forming anisotropic stacks, as shown in SEM images of graphite powder in ref. [53]. In addition, such stacks may orient during the shaping process parallel to the sample surface. The shape and orientation of the particle agglomerates was not quantified in the present study; however, an orientation of the graphite particles in the in-plane direction was described in previous studies [53,54]. In case of filler orientation in the in-plane direction, the thermal conductivity will also be higher in this direction and significantly lower when measured through the plane (the direction of measurement in our study). This indicates that the difference between the Hatta model and the measured values may be even higher when the measurement was done in the in-plane direction (not performed). Thus, the deviating shape of graphite from the spherical shape assumed in the model may be the main reason for the difference between calculated and measured values. 

Although the GNP are flake-shaped, they form agglomerates with a particle-like structure in the composites that is similar to graphite, as illustrated in ref. [54]. Thus, they were also considered in a simplified way as spherical particles. The comparison of the theoretical and experimental values is shown in Figure 3. Similar to graphite, also for this type of filler, the calculated values are lower than the experimentally determined thermal conductivity values. The difference between measured and calculated values is even higher than that for graphite. Again, the deviation of the real shape of GNP from the spherical shape assumption may be the main reason for this discrepancy showing the limitation of the Hatta model for fillers that are not spherical. Looking at Equation (1), it becomes clear that the thermal conductivity of the fillers λ_f_ at the used low filler loadings only contributes in a low extent to the thermal conductivity λ_c_ of the entire composite. Therefore, the calculated values for all the spherical particles (carbon black, GNP, and graphite) are quite the same, and are mainly determined by the polymer matrix conductivity. Differences in the shape and size cannot be reflected.

The use of the Hatta model for the calculation of the thermal conductivity of composites with carbon fibers or carbon nanotubes is not suitable, since the high aspect ratio of these fillers and thus the possible particle orientation in the test specimen is not taken into account. Therefore, the model of thermal conductivity developed by Xue [50] for carbon nanotube-based composites was chosen. In this model, the shape and the aspect ratio of the rod-like fillers, such as CNT and CF, are taken into consideration in the calculation, even if they do not explicitly appear in the equation:

(2) $$\lambda_{c}=\lambda_{m}\frac{1-V_{f}+2V_{f}\frac{\lambda_{f}}{\lambda_{f}-\lambda_{m}}ln\left(\frac{\lambda_{f}+\lambda_{m}}{2\lambda_{m}}\right)}{1-V_{f}+2V_{f}\frac{\lambda_{m}}{\lambda_{f}-\lambda_{m}}ln\left(\frac{\lambda_{f}+\lambda_{m}}{2\lambda_{m}}\right)}$$

where λ_c_, λ_m_, and λ_f_ are the thermal conductivities of the composite, matrix, and filler, respectively, and V_f_ is volume fraction of the filler.

The calculated thermal conductivities of composites filled with CF or CNTs predicted with Equation (2) are included in Figure 3 as blue dashed lines. For both composites filled with CF or CNT, the calculated thermal conductivity values are significantly higher than the measured values. The deviations of the values indicate that the model developed by Xue is slightly better suited for PP/CF composites than for PP composites filled with CNTs. This could be caused by the orientation of the fillers in the polymer matrix. As shown in Kunz et al. [53], the CNTs in a compression molded plate of polymer/CNT composite are mainly aligned in the plane. Due to this filler orientation, a higher thermal conductivity in-plane compared to through-plane can be expected. Xue [50] assumes in his model a random dispersion of the CNTs in the matrix in all spatial directions. Therefore, the deviations between the experimental measured values and the calculation can possibly be caused by the filler orientation. On the one hand, the filler orientation is opposite to the measuring direction and, on the other hand, the filler orientation in-plane is more pronounced than assumed in the Xue model.

### 3.2. Binary Filler Systems in PP

In the second part of the study, combinations of two different fillers were used, with a total filler content of 7.5 vol% being retained. The aim was to find the possible synergistic effects of the joint conductive networks of both fillers in the PP matrix concerning thermal conductivity and electrical volume resistivity. The measured values of the different binary systems are summarized in Table 3.

Firstly, combinations of the filler with the highest influence on thermal conductivity in PP, namely **GNP**, with the material leading to the most significant decrease in volume resistivity in PP, namely **CNT**, were investigated. The thermal conductivity achieved with PP/5 vol% GNP (0.39 W/(m·K)) can be further increased to 0.5 W/(m·K) (increase to 193% related to pure PP) by the addition of 2.5 vol% CNT. The same value of thermal conductivity was also achieved for the PP/7.5 vol% GNP composite, but both samples differ significantly in their electrical resistivity. The electrical volume resistivity at 7.5 vol% GNP loading was measured to be 3.3·10^6^ Ohm cm, while a value of t 5.1·10^2^ Ohm cm was measured for the GNP/CNT combination. At the lower GNP content of 2.5 vol% combined with 5 vol% CNT, a value of thermal conductivity of 0.46 W/(m·K) (177% related to pure PP) was measured, whereas the electrical resistivity was 1.9·10^2^ Ohm cm. These results indicate that GNP is more responsible for increasing the thermal conductivity and that the electrical properties are mainly influenced by the CNT content. Thus, synergistic effects could be observed for the combination of GNP and CNT both for the electrical resistivity and the thermal conductivity.

The combination of **GNP and CF** in PP was less effective at increasing the thermal conductivity or decreasing the electrical resistivity. The addition of 5 vol% CF to 2.5 vol% GNP in PP leads to a slight increase from 0.32 W/(m·K) (PP/2.5 vol% GNP) to 0.37 W/(m·K), whereas for a PP/5 vol% CF composite, the thermal conductivity was at the level of pure PP (compare Figure 3). Interestingly, the electrical resistivity of PP/5 vol% CF + 2.5 vol% GNP decreased to 1.0·10^8^ Ohm cm compared to PP/5 vol% CF or PP/2.5 vol% GNP, which are both electrical non-conductive (·10^17^ Ohm cm). For the PP/5 vol% GNP + 2.5 vol% CF composite, the thermal and electrical conductivities were not changed compared to PP/5 vol% GNP. Thus, synergistic effects could be observed for the combination of GNP and CF only for the electrical resistivity.

The combination of **GNP and G** in PP leads to the highest value for the thermal conductivity of 0.53 W/(m·K) (an increase of 204% compared to PP) when 5 vol% GNP and 2.5 vol% graphite were combined. The value of volume resistivity of both PP composites filled with GNP/G (2.5/5 and 5/2.5 vol%) indicated the formation of combined electrical networks. Although the PP/G composites were not electrically conductive up to 7.5 vol% G, the addition of G to PP/GNP leads to a reduction in the volume resistivity compared to the resistivity values of PP/GNP composites. The resistivity of PP/2.5 vol% GNP (1.0·10^17^ Ohm cm) and PP/5 vol% GNP (1.0·10^9^ Ohm cm) could be reduced to 5.9·10^9^ Ohm cm or 2.9·10^7^ Ohm cm, respectively, by the incorporation of 5 vol% G or 2.5 vol% G, respectively. Thus, synergistic effects could be observed for the combination of GNP and G only for the electrical resistivity.

For the combination of **GNP and CB**, high-structured (Printex^®^ XE2B) and low-structured CB (Printex^®^ P300) were applied. The results indicate that the use of higher structured CB leads to higher thermal conductivity and lower electrical resistivity. For the combination of 5 vol% GNP and 2.5 vol% XE2B, a thermal conductivity of 0.51 W/(m·K) was measured, which is one of the highest values in this series. A very low volume resistivity was found for PP/2.5 vol% GNP + 5 vol% XE2B, which was 9.2 Ohm cm. Thus, synergistic effects could be observed for the combination of GNP and CB both for the electrical resistivity and thermal conductivity.

The PP composites containing combinations of **G and CB** achieved low electrical volume resistivity values. For PP/5 vol% G + 2.5 vol% CB XE2B, an electrical resistivity of 47 Ohm cm was measured. Taking into account that PP/5 vol% G was non-conductive and a volume resistivity of 6.4·10^3^ Ohm cm was measured for PP/2.5% CB XE2B, there is a synergistic effect with regard to the electrical properties. For all the CB–G mixing ratios (see Table 3), the maximum thermal conductivity was 0.43 W/(m·K) (increase to 165% compared to PP), which does not represent an increase in thermal conductivity compared with the individual fillers.

The thermal conductivity of PP composites containing **CF and CNT** increased to 0.40 W/(m·K) compared to pure PP and thus to 154% compared to PP. Considering that PP composites filled only with CNTs or CF show no increase in thermal conductivity, this is a synergistic effect for thermal conductivity. The electrical volume resistivity of the PP/CF+CNT composites seems to be caused mainly by the presence of CNT, even if the value of PP with 2.5 vol% CNT (1.3·10^3^ Ohm cm) is slightly reduced by the addition of 5 vol% CF to 2.5·10^2^ Ohm cm.

### 3.3. Ternary Filler Systems in PP

The aim of using three fillers together in the PP matrix was to obtain a composite that combines the special properties of all the fillers. The incorporation of GNP in PP leads to a high thermal conductivity, and the CNT reduce the electrical resistivity of the PP composite. The third filler is intended to bridge the gaps in the combined conductive network of the two main fillers. For this purpose, on the one hand, a much larger filler (CF) was used, and on the other hand, a spherical filler was used (CB). The achieved values of thermal conductivity and electrical volume resistivity are included in Table 3.

For the ternary system, the highest thermal conductivity of 0.5 W/(m·K) and the lowest volume resistivity of 4.2 Ohm cm were obtained for the combination of **GNP, CNT, and highly structured CB** with 2.5 vol% of each filler. Since the thermal conductivity of the individual fillers at 2.5 vol% is 0.32 W/(m·K) for PP/GNP, 0.27 W/(m·K) for PP/CNT, and 0.33 W/(m·K) for PP/CB XE2B, there is a synergistic effect in the PP composite containing the three fillers. A synergistic effect can also be observed for the electrical resistivity of the PP/GNP+CNT+CB composite, because for PP/CNT and PP/CB, a volume resistivity of 1.3 10^3^ Ohm cm and 6.4 10^3^ Ohm cm, respectively, was measured at 2.5 vol%, and PP/2.5 vol% GNP was not electrically conductive. Both the large increase in thermal conductivity and the decrease of volume resistivity indicates the formation of a combined filler network of the three fillers within the PP matrix. 

With regard to the other ternary filler systems, the electrical resistivity value of the **PP/GNP+ CNT+ CF** composite was mainly influenced by the CNT addition and was found to be 5.5·10^2^ Ohm cm, which is only slightly lower than the value for PP/2.5 vol% CNT at 1.3 10^3^ Ohm cm. However, the combination of 2.5 vol% GNP + 2.5 vol% CNT + 2.5 vol% low-structured CB (P300) in PP leads also to a low value of volume resistivity of 9.3 Ohm cm. The result shows again that the high-structured CB (XE2B) is more suitable to decrease the volume resistivity than the low-structured CB (P300).

In summary, when fixing the filler content to 7.5 vol%, the best filler mixture in PP to achieve at the same time both high thermal and low electrical resistivity was the combination of GNP, CNT, and high-structured CB, each with 2.5 vol%.

### 3.4. Morphological Characterization

**Figure 4:**
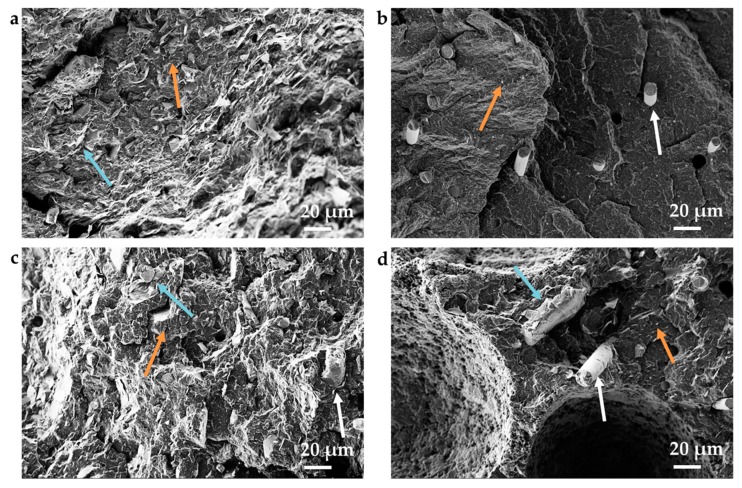
Scanning electron microscope images of cryofractured surfaces of PP composite strands filled with (**a**) 2.5 vol% CNT and 5 vol% GNP, (**b**) 5 vol% CNT and 2.5 vol% CF, (**c**,**d**) 2.5 vol% CF, 2.5 vol% CNT, and 2.5 vol% GNP (CNT: orange arrow, GNP: light blue arrow, CF: white arrow).

To illustrate the distribution of the fillers within the PP matrix, scanning electron microscopy on cryofractured surfaces was performed. In Figure 4, exemplarily polypropylene filled with 5 vol% GNP + 2.5 vol% CNT (Figure 4a), 5 vol% CNT + 2.5 vol% CF ( Figure 4b), as well as the combination of CF, CNT, and GNP with 2.5 vol% each (Figure 4c,d) are presented. All the fillers are well distributed. The carbon nanotubes are visible at the surfaces as small white points, which were especially seen in Figure 4b, and appear to be very well dispersed into separated tubes. The graphite nanoplatelets show thicknesses greater than those specified by the manufacturer, indicating that exfoliation was not fully successful under the chosen melt mixing conditions and graphitic-like stacks remained.

These images support the assumption that the combined filler networks between the used binary and ternary filler develop and that the fillers support each other in developing thermal and electrical conductivity.

## 4. Summary

For melt-mixed PP-based composites modified with different filler combinations of thermally and electrically conductive fillers, synergistic effects for thermal and electrical conductivity were found. Especially for the achievement of a high thermal conductivity, it seems to be advantageous to mix fillers with different shapes and aspect ratios to form a combined conductive network, as it was shown here for the combinations of GNP, G, CNT, CF, or CB. 

At a constant filler content of 7.5 vol%, the highest value of thermal conductivity was achieved with a combination of GNP (5 vol%) and graphite (2.5 vol%), which resulted in a value of 0.53 W/(m·K), which is more than twice the value of pure PP (0.26 W/(m·K)). 

For the electrical volume resistivity, synergistic effects were found if combinations of two or three fillers were used: PP filled with 2.5 vol% GNP and 5 vol% CF, PP/GNP+G composites, or PP/GNP or PP/G combined with high-structured CB. 

In summary, it was found that the thermal conductivity was mainly determined by the presence of graphite nanoplatelets, whereas the electrical resistivity was determined by the presence of multiwalled carbon nanotubes.

The PP composite having simultaneously high thermal (0.50 W/(m·K)) and low electrical resistivity (4.2 Ohm cm) contains a mixture of GNP, CNT, and high-structured CB with 2.5 vol% each.

The experimental results were supplemented by theoretical calculations on thermal conductivity. The Hatta model is suitable for PP containing spherical CB fillers, even if it is not able to reflect the differences between the differently structured carbon blacks. For PP composites containing graphite or graphite nanoplatelets, lower values for thermal conductivity were calculated with the Hatta model than those experimentally found. One reason for this may be the two-dimensional shape of the graphite flakes. For rod-like fillers such as CNTs and CFs, the Xue model was used to calculate the thermal conductivity. However, significantly higher thermal conductivity values were calculated for both fillers than measured experimentally. Therefore, these calculations are insufficient for the prediction. The cause is assumed to be the filler orientation in the polymer matrix.

## Figures and Tables

**Table 1:** Properties of various carbon blacks (data of supplier).

Printex Type	Surface Area (ISO 4652)	Oil Adsorption Number (OAN, ISO 4656)	Mean Aggregate Size	Average Primary Particle Size (ASTM D3849)
XE2B	1100 m²/g	410 mL/100g	910 nm	30 nm
L6	250 m²/g	123 mL/100g	250 nm	18 nm
300	77 m²/g	72 mL/100g	80 nm	27 nm

**Table 2:** Particle size of various kinds of graphite and carbon black.

Graphite	Mean Particle Size [µm]		
	x_10_	x_50_	x_90_
TIMCAL TIMREX® KS150	10	54	179
EP1200	70	124	205
TIMCAL TIMREX® KS500	23	144	416
Printex^®^ XE2B	5	60	365
Printex^®^ P300	2	11	128

**Table 3:** Thermal conductivity and electrical volume resistivity of PP composites containing 7.5 vol% carbon-based fillers (as Graphite KS500 was used), λ_pp_ = 0.26 W/(m·K). CB: carbon black, G: graphite.

GNP [vol%]	G [vol%]	CNT [vol%]	CF [vol%]	CB [vol%]	Thermal Conductivity λ_c_ [W/(m·K)]	λ_c/_λ_pp_ [%]	Electrical Resistivity [Ohm cm]
7.5	-	-	-	-	0.50	193	3.3·10^6^
-	7.5	-	-	-	0.47	180	4.0·10^16^
-	-	-	7.5	-	0.32	123	6.9·10^18^
-	-	-	-	7.5 (P300)	0.30	115	5.4·10^3^
5	-	2.5	-	-	0.50	193	5.1·10^2^
2.5	-	5	-	-	0.46	177	1.9·10^2^
5	-	-	2.5	-	0.40	154	1.5·10^9^
2.5	-	-	5	-	0.37	142	1.0·10^8^
5	2.5	-	-	-	0.53	204	2.9·10^7^
2.5	5	-	-	-	0.45	172	5.9·10^9^
5	-	-	-	2.5 (P300)	0.49	189	1.8·10^3^
2.5	-	-	-	5 (P300)	0.35	133	3.4·10^2^
5	-	-	-	2.5 (XE2B)	0.51	196	3.9·10^1^
2.5	-	-	-	5 (XE2B)	0.47	181	9.2·10^0^
-	5	-	-	2.5 (P300)	0.37	142	3.1·10^18^
-	2.5	-	-	5 (P300)	0.39	151	7.5·10^2^
-	5	-	-	2.5 (XE2B)	0.43	165	4.7·10^1^
-	-	2.5	5	-	0.40	154	8.3·10^2^
-	-	5	2.5	-	0.40	154	2.5·10^2^
2.5	-	2.5	2.5	-	0.44	169	5.5·10^2^
2.5	-	2.5	-	2.5 (P300)	0.45	172	9.3·10^0^
2.5	-	2.5	-	2.5 (XE2B)	0.50	193	4.2·10^0^

## Supplementary Materials

The following are available online at https://www.mdpi.com/2073-4360/11/6/1073/s1, Table S1: Thermal conductivity and electrical volume resistivity of PP composites filled with different kind of carbon black at a filler content of 5 vol%; Table S2: Thermal conductivity and electrical volume resistivity of PP composites filled with different kinds of expanded graphite at a filler content of 5 vol%; Table S3: Thermal conductivity and electrical volume resistivity of PP composites filled with different kinds of graphite at a filler content of 5 vol%.
