# Supplementary material for: Thermal Conductivity and Electrical Resistivity of Melt-Mixed Polypropylene Composites Containing Mixtures of Carbon-Based Fillers
Source: Polymers (Basel). 2019 Jun 21;11(6):1073. doi: 10.3390/polym11061073 (PMC6630210; doi:10.3390/polym11061073)
Supplement: Supplementary file 1 [file polymers-11-01073-s001.pdf]

Supplementary Materials: Table S1. Thermal conductivity and electrical volume resistivity of PP composites filled with different kind of carbon black at a filler content of 5 vol%; Table S2. Thermal conductivity and electrical volume resistivity of PP composites filled with different kinds of expanded graphite at a filler content of 5 vol%; Table S3. Thermal conductivity and electrical volume resistivity of PP composites filled with different kinds of graphite at a filler content of 5 vol%.

Table S1. Thermal conductivity and electrical volume resistivity of PP composites filled with different kind of carbon black at a filler content of 5 vol%

Filler name	Thermal conductivity [W/(m·K)]	Electrical resistivity [Ohm cm]	Manufacturer
XPB538	0.25	$1.64 \cdot 10^{2}$	Orion Engineered Carbons GmbH, Hanau, Germany
HIBlack 40B2	0.30	$1.29 \cdot 10^{2}$	Orion Engineered Carbons GmbH, Hanau, Germany
Grapacarb (LF-C)	0.26	$5.02 \cdot 10^{3}$	Harald Scholz & Co. GmbH, Recklinghausen, Germany
HS 0060	0.30	$2.16 \cdot 10^{2}$	Harald Scholz Co. GmbH, Recklinghausen, Germany
Lionite EC 200L	0.34	$1.74 \cdot 10^{1}$	LION Corporation, Tokyo, Japan
OMCARB C40	0.28	$2.04 \cdot 10^{17}$	Omsk Carbon Group, Omsk, Russia
OMCARB C140	0.29	$6.25 \cdot 10^{1}$	Omsk Carbon Group, Omsk, Russia
OMCARB CH85	0.30	$3.61 \cdot 10^{3}$	Omsk Carbon Group, Omsk, Russia
OMCARB CH200	0.29	$6.25 \cdot 10^{1}$	Omsk Carbon Group, Omsk, Russia
OMCARB CH210	0.31	$8.11 \cdot 10^{2}$	Omsk Carbon Group, Omsk, Russia
OMCARB CH600	0.28	$1.41 \cdot 10^{2}$	Omsk Carbon Group, Omsk, Russia
Mechano Cond 5P2	0.30	$8.11 \cdot 10^{2}$	H.C. Carbon GmbH, Rednitzhembach, Germany
Mechano Cond 5CP3	0.34	$4.42 \cdot 10^{14}$	H.C. Carbon GmbH, Rednitzhembach, Germany
Vulcan XC72 (VXC72)	0.29	$2.85 \cdot 10^{2}$	Cabot Corporation, Billerica, United States
BOTLEK VXC500	0.30	$7.79 \cdot 10^{1}$	Cabot Corporation, Billerica, United States
Ketjenblack EC300J	0.31	$1.10 \cdot 10^{1}$	AkzoNobel, Amsterdam, Netherlands
Ketjenblack EC600JD	0.34	$4.15 \cdot 10^{0}$	AkzoNobel, Amsterdam, Netherlands
ENSACO 150g (E150g)	0.31	$1.33 \cdot 10^{5}$	Imerys Graphite & Carbon, Bironico, Switzerland
ENSACO 250g (E250g)	0.31	$4.87 \cdot 10^{1}$	Imerys Graphite & Carbon, Bironico, Switzerland
TOKABLACK 4300	0.29	$5.18 \cdot 10^{17}$	Tokai Carbon Co., LTD, Tokyo, Japan
TOKABLACK 4400	0.29	$1.08 \cdot 10^{4}$	Tokai Carbon Co., LTD, Tokyo, Japan
TOKABLACK 4500	0.30	$1.53 \cdot 10^{5}$	Tokai Carbon Co., LTD, Tokyo, Japan
TOKABLACK 5500	0.30	$7.50 \cdot 10^{1}$	Tokai Carbon Co., LTD, Tokyo, Japan

Table S2. Thermal conductivity and electrical volume resistivity of PP composites filled with different kind of expanded graphite at a filler content of 5 vol%.

Filler name	Thermal conductivity [W/(m·K)]	Electrical resistivity [Ohm cm]	Manufacturer
GFG350	1.05	$7.71 \cdot 10^{4}$	SGL CARBON GmbH, Meitingen, Germany
GFG600	1.23	$8.12 \cdot 10^{10}$	SGL CARBON GmbH, Meitingen, Germany
GFG900	1.05	$1.15 \cdot 10^{2}$	SGL CARBON GmbH, Meitingen, Germany
GFG1000	0.66	$6.47 \cdot 10^{6}$	SGL CARBON GmbH, Meitingen, Germany
GFG1200	0.95	$1.40 \cdot 10^{2}$	SGL CARBON GmbH, Meitingen, Germany

Table S3 part 1. Thermal conductivity and electrical volume resistivity of PP composites filled with different kinds of graphite at a filler content of 5 vol%.

Filler name	Thermal conductivity [W/(m·K)]	Electrical resistivity [Ohm cm]	Manufacturer
EP1005	0.33	$6.35 \cdot 10^{8}$	Richard Anton KG, Gräfeling, Germany
SC150	0.51	$4.39 \cdot 10^{7}$	Graphit Kropfmühl GmbH, Hauzenberg, Germany
SC750	0.68	$4.55 \cdot 10^{4}$	Graphit Kropfmühl GmbH, Hauzenberg, Germany
GHL3292	0.40	$8.09 \cdot 10^{16}$	GEORG H. LUH GmbH, Walluf, Germany
Technographit, synth.	0.32	$1.14 \cdot 10^{9}$	TECHNOGRAFIT GmbH, Eltville, Germany
Thielmann, syn.Graphit	0.39	$1.10 \cdot 10^{9}$	Thielmann Graphite GmbH & Co. KG, Grolsheim, Germany
Mechano CAP1P1	0.30	$1.39 \cdot 10^{17}$	H.C. Carbon GmbH, Rednitzhembach, Germany
Mechano Cond 1P4	0.37	$3.84 \cdot 10^{12}$	H.C. Carbon GmbH. Rednitzhembach, Germany
Mechano Cond 1	0.30	$1.76 \cdot 10^{2}$	H.C. Carbon GmbH, Rednitzhembach, Germany
Grafit GS 120	0.36	$4.20 \cdot 10^{10}$	RMC REMACON GmbH, Säckingen, Germany
Grafit LUX Carbon 99G	0.34	$2.77 \cdot 10^{16}$	LUX Carbon Rohstoffhandelsgesellschaft, Essen, Germany
Grafit RFL99.5	0.40	$1.54 \cdot 10^{13}$	Graphit Kropfmühl GmbH, Hauzenberg, Germany
Graphit TG 100/400	0.30	$6.87 \cdot 10^{10}$	Mineralmühle Leun, Rau GmbH & Co. KG, Leun, Germany

Table S3 part 2. Thermal conductivity and electrical volume resistivity of PP composites filled with different kinds of graphite at a filler content of 5 vol%.

Filler name	Thermal conductivity [W/(m·K)]	Electrical resistivity [Ohm cm]	Manufacturer
FGM99	0.40	$2.50 \cdot 10^{16}$	Grafitbergbau Kaiersberg Ges.m.b.H., St. Stefan ob Leoben, Austria
Thielmann Flockengraphit 22003	0.42	$1.52 \cdot 10^{16}$	Thielmann Graphite GmbH & Co. KG, Grolsheim, Germany
Technographit großkristallin Natur 70-55	0.39	$4.68 \cdot 10^{16}$	TECHNOGRAFIT GmbH, Eltville Germany
(LUH40) LUH Synthetischer Graphit min. 99.3%	0.33	$1.22 \cdot 10^{17}$	TECHNOGRAFIT GmbH, Eltville Germany
(LUH60) LUH Synthetischer Graphit min. 99.3%	0.33	$3.54 \cdot 10^{16}$	TECHNOGRAFIT GmbH, Eltville Germany
(LUH80) LUH Synthetischer Graphit min. 99.3%	0.34	$1.46 \cdot 10^{17}$	TECHNOGRAFIT GmbH, Eltville Germany
(LUH100) LUH Synthetischer Graphit min. 99.3%	0.33	$1.27 \cdot 10^{17}$	TECHNOGRAFIT GmbH, Eltville Germany
TIMCAL TIMREX BNB 90	0.81	$5.69 \cdot 10^{7}$	Imerys Graphite & Carbon, Bironice, Switzerland
Timrex KS150-600	0.42	$5.92 \cdot 10^{7}$	Imerys Graphite & Carbon, Bironice, Switzerland
Timrex KS75	0.36	$1.24 \cdot 10^{16}$	Imerys Graphite & Carbon, Bironice, Switzerland
Timrex T150-600	0.39	$9.16 \cdot 10^{6}$	Imerys Graphite & Carbon, Bironice, Switzerland
Timrex T150	0.32	$3.91 \cdot 10^{14}$	Imerys Graphite & Carbon, Bironice, Switzerland
Asbury TC300	0.39	$3.42 \cdot 10^{7}$	Asbury Graphite Mills, Inc., New Jersey, United States
Asbury 4012	0.32	$1.54 \cdot 10^{12}$	Asbury Graphite Mills, Inc., New Jersey, United States
Asbury 94002	0.28	$1.76 \cdot 10^{8}$	Asbury Graphite Mills, Inc., New Jersey, United States
Remacon GS12	0.40	$4.37 \cdot 10^{17}$	RMC REMACON GmbH. Bad Säckingen. Germany

Table S3 part 3. Thermal conductivity and electrical volume resistivity of PP composites filled with different kind of graphite at a filler content of 5 vol%.

Filler name	Thermal conductivity [W/(m·K)]	Electrical resistivity [Ohm cm]	Manufacturer
Remacon GS40	0.39	$1.07 \cdot 10^{17}$	RMC REMACON GmbH, Bad Säckingen, Germany
SGL Sigrafine V1032	0.29	$3.93 \cdot 10^{17}$	SGL Carbon, Wiesbaden, Germany
Kropfmühl SGA20	0.43	$1.44 \cdot 10^{11}$	Graphit Kropfmühl GmbH, Hauzenberg, Germany
Kropfmühl SC20OS	0.55	$9.41 \cdot 10^{6}$	Graphit Kropfmühl GmbH, Hauzenberg, Germany
